# Transmission of *Tomato Yellow Leaf Curl Virus* by *Bemisia tabaci* as Affected by Whitefly Sex and Biotype

**DOI:** 10.1038/srep10744

**Published:** 2015-05-29

**Authors:** Wenxi Ning, Xiaobin Shi, Baiming Liu, Huipeng Pan, Wanting Wei, Yang Zeng, Xinpei Sun, Wen Xie, Shaoli Wang, Qingjun Wu, Jiaxu Cheng, Zhengke Peng, Youjun Zhang

**Affiliations:** 1 College of Life Science, Northeast Agricultural University, Harbin, P. R. China; 2 Department of Plant Protection, Institute of Vegetables and Flowers, Chinese Academy of Agricultural Sciences, Beijing, P. R. China; 3 Institute of Plant Protection, Tianjin Academy of Agricultural Sciences, Tianjin, P. R. China

## Abstract

*Bemisia tabaci* is a serious pest of vegetables and other crops worldwide. The most damaging and predominant *B. tabaci* biotypes are B and Q, and both are vectors of *tomato yellow leaf curl virus* (TYLCV). Previous research has shown that Q outperforms B in many respects but comparative research is lacking on the ability of B and Q to transmit viruses. In the present study, we tested the hypothesis that B and Q differ in their ability to transmit TYLCV and that this difference helps explain TYLCV outbreaks. We compared the acquisition, retention, and transmission of TYLCV by B and Q females and males. We found that Q females are more efficient than Q males, B females, and B males at TYLCV acquisition and transmission. Although TYLCV acquisition and transmission tended to be greater for B females than B males, the differences were not statistically significant. Based on electrical penetration graphs determination of phloem sap ingestion parameters, females fed better than males, and Q females fed better than Q males, B females, or B males. These results are consistent with the occurrences of TYLCV outbreaks in China, which have been associated with the spread of Q rather than B.

*Tomato yellow leaf curl virus* (TYLCV, genus *Begomovirus*, family *Geminiviridae*) is a global threat to agriculture^1^. TYLCV originated in the Middle Eastern-Mediterranean region, and since then has spread to many regions of the world^2^,^3^. TYLCV is transmitted by the whitefly *Bemisia tabaci* (Gennadius) (Hemiptera: Aleyrodidae) in a persistent and circulative manner^4^,^5^.

*B. tabaci* is considered to be a complex of at least 24 biotypes that are difficult or impossible to distinguish based on morphology. *B. tabaci* B (which originated in the Middle East**-**Minor Asia) and *B. tabaci* Q (which originated in the Mediterranean region) are the two most invasive and destructive whiteflies^6^. *B. tabaci* B and Q (hereafter simply B and Q) differ in host range, feeding behavior, endosymbionts, insecticide resistance, and virus transmission efficiency^7^,^8^,^9^,^10^. Both B and Q seriously damage plants by feeding on phloem and by transmitting plant viruses.

In China, TYLCV was first detected almost simultaneously with the invasion by Q^11^,^12^. Liu *et al.* (2013)^10^ found that TYLCV-infected plants alter whitefly feeding behavior so as to benefit Q but harm B. Pan *et al.* (2012)^12^ reported that Q performed better but B performed worse on TYLCV-infected tomato plants than on non-infected tomato plants. Shi *et al.* (2014)^13^ showed that JA-related plant defenses were reduced more by viruliferous Q than by viruliferous B. These findings showed that fitness was greater for Q than B when feeding on TYLCV-infected plants.

Information on the ability of vectors to acquire, retain, and transmit a virus is critical for understanding and managing virus outbreaks^14^. Although the acquisition, retention, and transmission of TYLCV by *B. tabaci* have been extensively examined^14^,^15^,^16^,^17^, virus transmission by B and Q has seldom been compared. Using quantitative real-time polymerase chain reaction (q-PCR), Pan *et al.* (2012)^12^ found that Q acquired significantly more TYLCV DNA than B. Comparative, detailed research on the transmission characteristics of B and Q, however, is still lacking.

Transmission of TYLCV is affected by *B. tabaci* gender. Previous results showed that TYLCV can be transmitted from insect to insect in a sex-dependent manner^16^. Xie *et al.* (2012)^18^ also showed that sex affected TYLCV transmission efficiency in *B. tabaci*. No previous study, however, has compared the TYLCV transmission efficiency of B and Q females and males. Such information will enhance the understanding and therefore the management of TYLCV outbreaks.

In the present study, we used q-PCR to systematically and specifically compare acquisition, retention, and transmission of TYLCV by both sexes of B and Q. We also compared the feeding behavior of females and males of B and Q. Our results will provide an overall reference to effectively monitor and manage TYLCV and its vectors.

## Results

### Acquisition of TYLCV DNA

**Figure 1:**
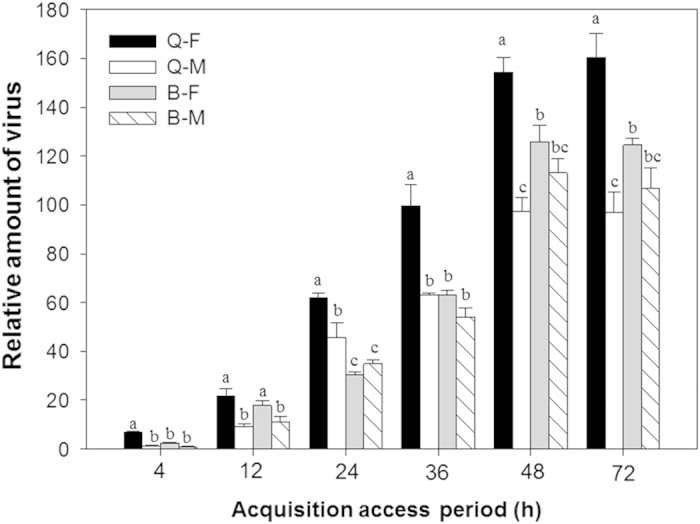
Acquisition of TYLCV DNA by males and females of B and Q *Bemisia tabaci*. Q-F: Q females; Q-M: Q males; B-F: B females; B-M: B males. Values are means ± SE. Means with different letters are significantly different (LSD test at P < 0.05).

TYLCV acquisition differed among the four combinations of *B. tabaci* biotype and sex (*F*_3, 8_ = 46.112, *P* < 0.001). Q females acquired significantly more virus than the other three combinations of sex and biotype (Fig. 1). TYLCV acquisition did not differ, however, between B females and B males. For all combinations, TYLCV DNA attained maximal viral loads in whiteflies after a 48-h acquisition access period.

### Retention of TYLCV DNA

**Figure 2:**
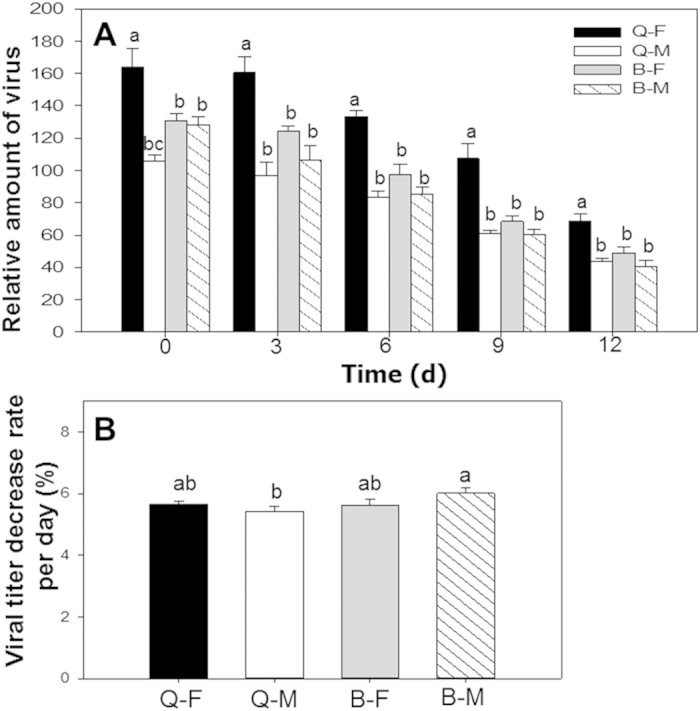
Retention of TYLCV DNA by males and females of B and Q *Bemisia tabaci* after transfer to non-host (cotton) plants. **A** Relative quantity of TYLCV retained per whitefly. **B** The rate at which TYLCV titer in whiteflies decreased. Q-F: Q females; Q-M: Q males; B-F: B females; B-M: B males. Values are means ± SE. For each time after transfer to cotton, means with different letters are significantly different (LSD test at P<0.05).

As indicated by q-PCR, the relative amount of TYLCV was greater in Q females than in the other three combinations on day 0, when the whiteflies were transferred to cotton plants (Fig. 2A). The titer decreased gradually in all combinations of sex and biotype but was always greater in Q females than in the other combinations (Fig. 2A). The rate at which the titer declined was greater for B males than Q males and was intermediate for B and Q females (Fig. 2B).

### Transmission of TYLCV

**Figure 3:**
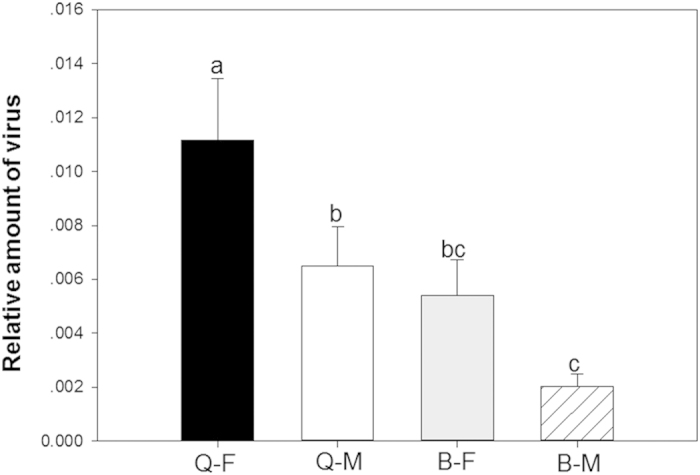
Transmission of TYLCV DNA by males and females of B and Q *Bemisia tabaci*. Q-F: Q females; Q-M: Q males; B-F: B females; B-M: B males. Values are means ± SE. Means with different letters are significantly different (LSD test at P<0.05).

Transmission of TYLCV, as indicated by the relative amount of virus in tomato leaves exposed to one whitefly, was affected by the whitefly sex (*F*_1, 36_ = 7.053, *P* = 0.012), the whitefly biotype (*F*_1, 36_ = 11.262, *P* = 0.002), and the interaction between these two factors (*F*_1, 36_ = 1.90, *P* = 0.666). Transmission was significantly higher for Q females than for the other three combinations of sex and biotype (Fig. 3).

### Feeding behavior of *B. tabaci*

**Figure 4:**
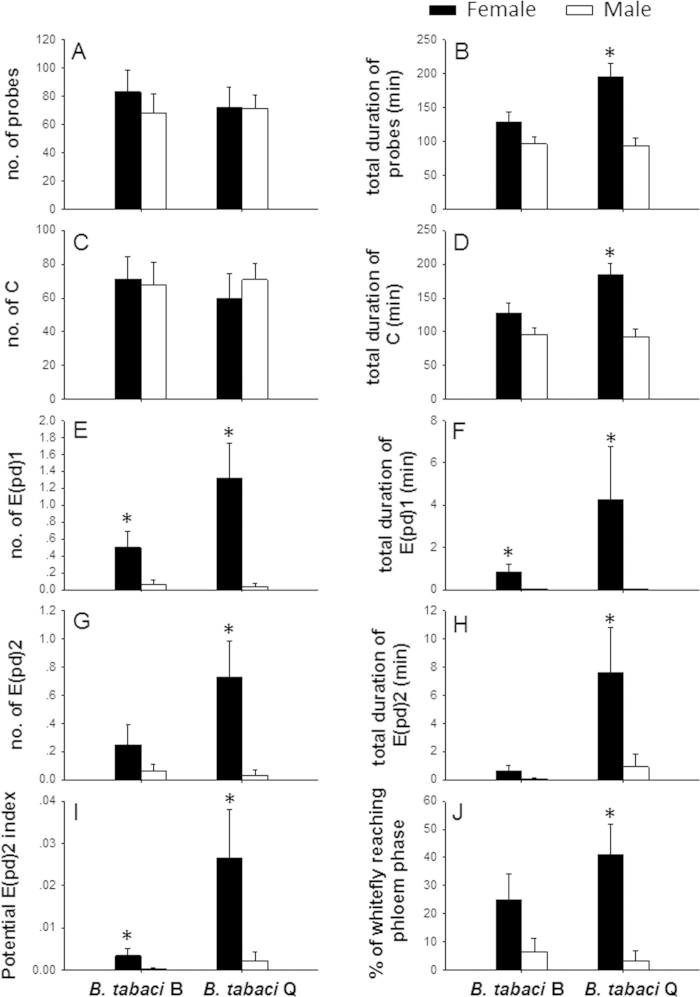
Feeding behavior of males and females of B and Q *Bemisia tabaci*. **A** total number of probes. **B** time of total probes. **C** total number of pathway probes. **D** total time of pathway probes. **E** total number of salivation into phloem. **F** total time of salivation. **G** total number of phloem ingestion. **H** total time of phloem ingestion. **I** (total time in E(pd)2)/(total recording time minus time to first E(pd)). **J** whitefly which reaching phloem phrase/all whiteflies tested in the treatment. All parameters were determined in 8 hrs EPG records time. Values are means ± SE. An asterisk indicates a significant difference between females and males within the indicated biotype (**A**–**I**: Mann-Whitney test and **J**: χ^2^ test at *P* < 0.05).

We calculated four non-phloem parameters (Fig. 4A-D) and six phloem parameters (Fig. 4E-J) in our EPG analysis. For B, the number of salivation probes was 6.5-times greater and salivation lasted 21.9-times longer for females than for males (Fig. 4E and F). Also, the Potential E(pd)2 index was 8.7-times greater for B females than for B males (Fig. 4I). However, the four non-phloem parameters did not significantly differ between males of females of B (Fig. 4A-D). For Q, only two non-phloem parameters of the 10 EPG parameters did not significantly differ between males and females (Fig. 4A and B). The total duration of probes was 1.1-time greater and the duration of pathway probes was 0.97-times greater for Q females than Q males (Fig. 4B and D). The number of salivation probes was 37.2-times greater and their total duration was 173-times greater for Q females than Q males (Fig. 4E and F). For phloem sap ingestion, the number of probes was 20.1-times greater and lasted 7.2-times longer for Q females than for Q males (Fig. 4G and H). The Potential E(pd)2 index was 11-times higher for Q females than for Q males, and the percentage of whiteflies that achieved phloem feeding was 10.9-times greater for Q females than for Q males (Fig. 4I and J).

## Discussion

Our results indicate that the efficiency of acquiring and transmitting TYLCV by *B. tabaci* biotypes was much higher for Q females than for Q males, B females, or B males. Previous results showed that Q represented about 78% of the *B. tabaci* biotypes detected in 18 provinces in China^12^. Our findings are consistent with the view that the outbreak and spread of TYLCV is closely related to an increase in the abundance and spread of Q rather than B in the field^12^.

We found that the ability to acquire TYLCV was greater for Q females than for B females. This result is in accord with a previous report that Q acquired more TYLCV than B^19^. Furthermore, we found that the ability to acquire TYLCV was significantly affected by the sex within each biotype. Q females acquired much more virus than Q males. One possible explanation for this difference in the quantity of virus acquired is that *B. tabaci* females are much larger than *B. tabaci* males^20^, but the relationship between *B. tabaci* size and its acquisition ability still needs to be confirmed.

After viruliferous whiteflies were transferred to cotton, which is not a host of TYLCV, the TYLCV titer in the whiteflies gradually decreased. Although the rate of decrease did not substantially differ between B and Q, the quantity of virus retained was much higher in Q females than in Q males, B females, or B males. It follows that Q females retain a greater quantity of TYLCV than the other combinations of biotype and sex because they acquire a greater quantity of TYLCV.

We also found that the quantity of TYLCV in tomato, which is a host plant for the virus, was greater when plants were infested with viruliferous Q females than with viruliferous Q males, B females, or B males. This indicates that Q females transmit more virus than the other combinations of *B. tabaci* sex and biotype to host plants.

Our results are generally consistent with those obtained for other insects and viruses in that many insect vectors transmit viruses in a sex-dependent manner, and transmission ability is usually greater for female than for male vectors. For example, females of the planthopper *Peregrinus maidis* are more efficient vectors of *rice stripe virus* than males^21^. However, female insects are sometimes less effective vectors than male insects. Male thrips, for example, transmit virus more efficiently than female thrips^22^,^23^. In our study, TYLCV transmission efficiency did not significantly differ between B females and B males but tended to be greater for B females, which is consistent with Xie *et al.* (2012)^18^. Although the difference was not significant, TYLCV transmission efficiency was two- to three-time greater for B females than for B males; this difference may have attained statistical significance with increased replication.

Our EPG data showed that the females feed better than males regardless of biotype (Fig. 4A-J). In particular, Q females salivated more times and for longer times than Q males, B females, or B males (Fig. 4E and F). Phloem sap ingestion parameters were greater for females and especially for Q females than for B or Q males (Fig. 4G and J). Because phloem salivation and sap ingestion behaviors are closely related to virus injection and virus ingestion, respectively^24^, our EPG data helps explain why virus transmission capability is much greater for females than for males. Our EPG results also confirmed that the virus transmission capability of the B and Q biotypes of *B. tabaci* is highest for Q females, which is consistent with our results that Q females are more efficient than Q males, B females, or B males at TYLCV acquisition and transmission.

Although the feeding behavior documented here helps explain why Q and B females may transmit virus more efficiently than Q and B males, this difference between the sexes can also have other explanations. Chiykowski (1967)^25^ proposed that females of the aster leafhopper must spend more time feeding than males because they support ovarial development and because they are generally larger than males^26^. An increase in feeding time would result in increased opportunities for females to acquire virus from infected plants and to transmit virus to healthy plants. Another possible explanation for the effect of vector sex on virus transmission could involve endosymbionts. Guo *et al.* (2014)^27^ reported that obligate symbionts and TYLCV occurred with greater frequency in *B. tabaci* females than males. Pan *et al.* (2012)^9^ showed that, among field populations of B and Q, the frequency of infection by the endosymbiont *Hamiltonella* sp. was higher in females than in males. Additional research is needed to determine whether endosymbionts affect the abilities of B and Q to acquire and transmit TYLCV. Other factors that can affect sex ratios in the field may also contribute to the more efficient transmission of female than males.

Our results also indicate that the capacity to acquire and transmit TYLCV is much greater for Q than B regardless of sex. In the EPG results Q males and B males showed no difference in feeding behavior, but Q males inoculated higher virus in plants than B males (Fig. 3). Previous research documented greater feeding by viruliferous Q than viruliferous B, which seems likely to increase TYLCV transmission rates^10^. One possible reason is that TYLCV may change plant chemistry in ways that favor Q over B. In addition, we previously reported that the ability to reduce the plant signaling defense response was much greater for viruliferous Q than for viruliferous B^28^,^29^. Some other aspects of the Q-host plant interaction may also favor accumulation of virus titer in plants. These results are consistent with view that the rapid replacement of B by Q throughout China is closely related to TYLCV.

In conclusion, our results suggest that the spread of TYLCV in the field is likely to be affected by *B. tabaci* biotype. More specifically, transmission is likely to increase with the abundance of Q rather than B because Q females are efficient vectors of TYLCV. We also found that TYLCV acquisition and transmission were much higher for Q females than for Q males and that this difference is related to feeding behavior, i.e., feeding is greater for Q females than for Q males. Our findings are consistent with previous reports concerning the nearly simultaneous spread of TYLCV and Q in China^12^. More research is needed to determine the mechanisms that cause the differences in TYLCV transmission efficiency among Q and B females and males of *B. tabaci*.

## Methods

### Insects and plants

The B population of *B. tabaci* was initially collected from cabbage, *Brassica oleracea L*. cv. Jingfeng 1, in Beijing, China, in 2004^30^, and the Q population was initially collected from poinsettia, *Euphorbia pulcherrima* Wild. ex Klotz., in Beijing, China, in 2009. After they were collected, the B and Q whiteflies were separately reared on healthy tomato plants, *Solanum lycopersicum* Mill. cv. Zhongza 9, in whitefly-proof screen cages in a greenhouse at 26 ± 2 ^o^C. The purities of the B and Q populations were monitored by sampling 15 adults per generation using a molecular diagnostic technique (CAPS: cleavage amplified polymorphic sequence) and the mitochondrial cytochrome oxidase I genes: *mtCOI*^31^.

Healthy and TYLCV-infected tomato plants were used. Tomato plants at the three-true-leaf stage were injected with a cloned TYLCV genome (GenBank accession number: AM282874), which was isolated from tomato plants in Shanghai, China. Infection of inoculated plants was confirmed by the appearance of typical leaf curl symptoms^32^.

### Acquisition of TYLCV by whiteflies

After they had been starved for 2 h, about 500 newly emerged whiteflies were released into each of three replicate cages per whitefly biotype (B and Q); each cage contained one TYLCV-infected tomato plant. After acquisition access periods (AAPs) of 4, 12, 24, 36, 48, and 72 h, adults were collected randomly from symptomatic leaves in each cage and were separated by sex. Then, 20 females and 20 males per replicate cage of both B and Q were stored at −80 ^o^C; the content of TVLCV DNA in these whiteflies was assessed by q-PCR as described later.

### Retention of TYLCV DNA by whiteflies

Approximately 500 newly emerged B or Q adults were released into each of three replicate cages containing one TYLCV-infected tomato plant. After a 72-h AAP, all 500 adults were transferred to each of three cages containing one cotton plant; cotton is not a host of TYLCV. Adults were randomly collected from each cage after 0, 3, 6, 9, and 12 d and separated by sex. Then, 20 females and 20 males per replicate cage of both B and Q were stored at −80 ^o^C; the content of TVLCV DNA in these whiteflies was assessed by q-PCR as described later.

### Transmission of TYLCV by whiteflies

Newly emerged B and Q adults were placed on TYLCV-infected tomato plants in cages. After a 72-h AAP, adults were collected and placed in clip-cages attached to healthy tomato plants with three true leaves; each plant had one clip cage that contained one adult. Each of four treatments (one Q female, one Q male, one B female, and one B male) was represented by 10 replicate clip cages. After the clip cages had been in place for 48 h, all of the whiteflies were removed, and the plants were kept in insect-proof cages. After 10 d, the first two youngest leaves of each plant were collected^33^,^34^; the DNA in the leaves was extracted and assessed for TYLCV by q-PCR as described in the next section.

### Detection of TVLCV DNA by q-PCR

For quantification of TYLCV acquisition and retention, the DNA in B and Q females and males was extracted using the MiniBEST universal genomic DNA extraction kit ver.5.0, Cod: 9765 (TaKaRa Biotechnology, Dalian Co., Ltd.). For quantification of TYLCV in plants, DNA was extracted from leaves with a plant genomic DNA kit (Tiangen Biotech, Beijing, Co., Ltd.). TYLCV DNA was quantified with SuperReal PreMix Plus (SYBR Green) (Tiangen Biotech). The primers used for quantification of TYLCV are listed in Table 1^18^. The q-PCR reaction conditions were previously described by Pan *et al.* (2012)^12^. For each sample, four technical replicates were amplified. The relative quantities of TYLCV were calculated based on the comparative cycle threshold 2^−ΔCt^ method.

### EPG experiment

We assessed the feeding behavior of Q females, Q males, B females, and B males of *B. tabaci*. The whiteflies were allowed to feed on healthy tomato plants at the 3-true-leaf stage. Each of the four combinations of biotype and sex was initially represented by 30 replicate whiteflies. However, experimental difficulties (e.g., whiteflies escaping or dying or probes becoming detached) resulted in slightly different levels of replication among the four groups of whiteflies; replicate number ranged from 22 to 30. The feeding behavior of each replicate whitefly was recorded for 8 h.

### EPG recoding

Whitefly feeding behavior was recorded by electrical penetration graphs using a DC-system (a Giga-8 DC-amplifier with a 10^9^ –Ω input resistance, Wageningen University, Netherlands). Before recoding, whiteflies were immobilized by cooling on a glass dish resting on an ice-pack. Then, a gold wire (1.5-cm × 12.5-μm) was attached to the whitefly’s dorsum using a drop of water-based silver glue. Each wired whitefly was connected to the Giga-8 probe input and then placed on the lower surface of the bottom leaf of a tomato plant (one whitefly per leaf and per plant). The whitefly, plant, and probe were placed in a Faraday cage (one whitefly per cage) to shield against electrical noise. Signals generated by whitefly feeding were digitized with a DI710-UL analogue-to-digital converter (Dataq Instruments, Akron, OH), and the output was acquired and stored with PROBE3.4 software (Wageningen University, Netherlands). All experiments were carried out at 26 ± 2 ^o^C and with 70% relative humidity.

### Data analysis

Waveforms of EPG were identified according to previous classification patterns^24^,^35^,^36^. The phloem EPG parameters were statistically analysed as reported by Liu *et al.* (2013)^10^. Mann-Whitney test (A-I) and χ^2^ test (J) at *P* < 0.05 were used to compare means of EPG parameters. SPSS 17.0 (SPSS Inc., Chicago, IL, USA) was used for statistical analysis. Two-way analyses of variance (ANOVAs) and repeated-measures ANOVAs were used to compare acquisition, retention, and transmission among B and Q females and males. Means were compared by least significant difference (LSD) tests at *P* < 0.05.

## Additional Information

**How to cite this article**: Ning, W. *et al.* Transmission of *Tomato Yellow Leaf Curl Virus by Bemisia tabaci* as affected by whitefly sex and biotype. *Sci. Rep.* **5**, 10744; doi: 10.1038/srep10744 (2015).

## Figures and Tables

**Table 1:** Primer sequences used for q-PCR analysis.

**Gene**	**Primer**	**Primer sequence (5’to 3’)**
**TYLCV**	TY-F	GTCTACACGCTTACGCC
	TY-R	GCAATCTTCGTCACCC
***Bemisia tabaci***	Actin-F	CGCTGCCTCCACCTCATT
	Actin-R	ACCGCAAGATTCCATACCC
	EF-1αF	TAGCCTTGTGCCAATTTCCG
	EF-1αR	CCTTCAGCATTACCGTCC
**tomato**	Actin-F	GGAAAAGCTTGCCTATGTGG
	Actin-R	CCTGCAGCTTCCATACCAAT
	UBI-F	TCGTAAGGAGTGCCCTAATGCTGA
	UBI-R	CAATCGCCTCCAGCCTTGTTGTAA
